# Uptake of a Consumer-Focused mHealth Application for the Assessment and Prevention of Heart Disease: The <30 Days Study

**DOI:** 10.2196/mhealth.4730

**Published:** 2016-03-24

**Authors:** Shivani Goyal, Plinio P Morita, Peter Picton, Emily Seto, Ahmad Zbib, Joseph A Cafazzo

**Affiliations:** ^1^ Centre for Global eHealth Innovation Techna Institute University Health Network Toronto, ON Canada; ^2^ Institute of Biomaterials and Biomedical Engineering University of Toronto Toronto, ON Canada; ^3^ Institute of Health Policy, Management and Evaluation University of Toronto Toronto, ON Canada; ^4^ Heart and Stroke Foundation of Canada Toronto, ON Canada

**Keywords:** health behavior, lifestyle, cardiovascular disease, prevention, risk reduction, mobile apps, mobile phone, incentives

## Abstract

**Background:**

Lifestyle behavior modification can reduce the risk of cardiovascular disease, one of the leading causes of death worldwide, by up to 80%. We hypothesized that a dynamic risk assessment and behavior change tool delivered as a mobile app, hosted by a reputable nonprofit organization, would promote uptake among community members. We also predicted that the uptake would be influenced by incentives offered for downloading the mobile app.

**Objective:**

The primary objective of our study was to evaluate the engagement levels of participants using the novel risk management app. The secondary aim was to assess the effect of incentives on the overall uptake and usage behaviors.

**Methods:**

We publicly launched the app through the iTunes App Store and collected usage data over 5 months. Aggregate information included population-level data on download rates, use, risk factors, and user demographics. We used descriptive statistics to identify usage patterns, *t* tests, and analysis of variance to compare group means. Correlation and regression analyses determined the relationship between usage and demographic variables.

**Results:**

We captured detailed mobile usage data from 69,952 users over a 5-month period, of whom 23,727 (33.92%) were registered during a 1-month AIR MILES promotion. Of those who completed the risk assessment, 73.92% (42,380/57,330) were female, and 59.38% (34,042/57,330) were <30 years old. While the older demographic had significantly lower uptake than the younger demographic, with only 8.97% of users aged ≥51 years old downloading the app, the older demographic completed more challenges than their younger counterparts (*F*_8, 52,422_ = 55.10, *P*<.001). In terms of engagement levels, 84.94% (44,537/52,431) of users completed 1–14 challenges over a 30-day period, and 10.03% (5,259/52,431) of users completed >22 challenges. On average, users in the incentives group completed slightly more challenges during the first 30 days of the intervention (mean 7.9, SD 0.13) than those in the nonincentives group (mean 6.1, SD 0.06, *t*_28870_=–12.293, *P*<.001, *d*=0.12, 95% CI –2.02 to –1.47). The regression analysis suggested that sex, age group, ethnicity, having 5 of the risk factors (all but alcohol), incentives, and the number of family histories were predictors of the number of challenges completed by a user (*F*_14, 56,538_ = 86.644, *P*<.001, adjusted *R* ^2^ = .021).

**Conclusion:**

While the younger population downloaded the app the most, the older population demonstrated greater sustained engagement. Behavior change apps have the potential to reach a targeted population previously thought to be uninterested in or unable to use mobile apps. The development of such apps should assume that older adults will in fact engage if the behavior change elements are suitably designed, integrated into daily routines, and tailored. Incentives may be the stepping-stone that is needed to guide the general population toward preventative tools and promote sustained behavior change.

## Introduction

Heart disease and stroke remain the leading causes of death and disability worldwide, and are responsible for almost 30% of all deaths [1]. The majority of the world’s adult population has at least one modifiable risk factor for cardiovascular disease (CVD), such as obesity, hypertension, physical inactivity, poor nutrition, and tobacco or alcohol consumption [2]. As with many chronic diseases, modifiable risk factors can be prevented by (1) identifying unhealthy lifestyle behaviors and (2) providing education and support to guide individuals toward behavior change and subsequent lifestyle modification [3,4].

Although health risk assessments have been an effective approach for health promotion and risk projection, the increased awareness of risk does not necessarily translate into behavior change [4,5]. Existing paper and Internet-based assessments are one-dimensional and do not provide patients with the tools and education required to actively work toward reducing their cardiovascular risk on a day-to-day basis [6]. For example, Heart Aware [3], an Internet-based cardiac risk assessment deployed to 373,085 users, provided an overview of risk based on the information that users entered. However, it did not provide continued guidance or actionable knowledge to enable individuals to work toward reducing their risk [3].

While many workplace wellness programs do offer tools with components of self-education and counseling that target and effectively reduce chronic diseases, they are not scalable to larger community-based populations [7]. These programs are typically adopted by large organizations that have the resources and infrastructure needed to successfully implement and maintain such programs. They do not focus on developing capacity among community members, which is necessary to support, implement, and sustain an effective preventive program [8].

Engaging individuals in risk factor identification and modification is crucial for preventing CVD. However, given the high prevalence of CVD, it is clear that current primary preventive actions are suboptimal [9]. Technology, such as mobile phones, can enable people to gain access to health promotion resources and peers within their community [10]. The increasing market penetration of mobile phones presents a unique opportunity not only to deliver evidence-based dynamic health risk assessments, but also to promote lifestyle modification interventions [10].

With this premise, we hypothesized that a dynamic risk assessment delivered as a mobile phone app, hosted by a reputable public nonprofit organization, would promote uptake among community members. The Heart and Stroke Foundation of Canada commissioned the development and public deployment of <30 Days, a mobile CVD risk assessment and management app. We hypothesized that the uptake would be influenced by the incentives offered for downloading the mobile app. We collected usage data at a population level to provide insight on uptake and engagement levels.

The goal of our study was to assess levels of engagement in terms of number of challenges completed and duration of use, and to compare usage and uptake between the incentives group and the nonincentives group. We anticipated that the users from the incentives group would be initially attracted to the app but would subsequently have lower levels of engagement than the nonincentives group.

## Methods

The Heart and Stroke Foundation of Canada launched the app on the iTunes App Store (Apple, Inc, Cupertino, CA, USA) and collected population-level usage data over 5 months. We obtained consent to analyze de-identified usage data for research purposes from users in the form of an in-app mobile agreement. Aggregate data included information on download rates, usage, feedback, and demographics of users around the globe.

### Mobile App Overview

The aim of the <30 Days app was to empower users to easily and effectively manage their heart health on a daily basis. Principles of user-centered design guided the conceptualization of the app, where feedback from end users informed the concept and key features [11]. In addition to the requirements gathered from users, we used constructs from the theory of planned behavior to guide the overall structure and motivations of the app [12]. The resulting app was available for download in Canada, free of charge, on the iTunes App Store. On downloading the app, users had the option either to complete the risk assessment right away or to temporarily skip the risk assessment and preview the app content. Users who opted to browse the app first were only offered 3 sample challenges before they were required to complete the risk assessment in order to continue using the app.

**Figure 1:**
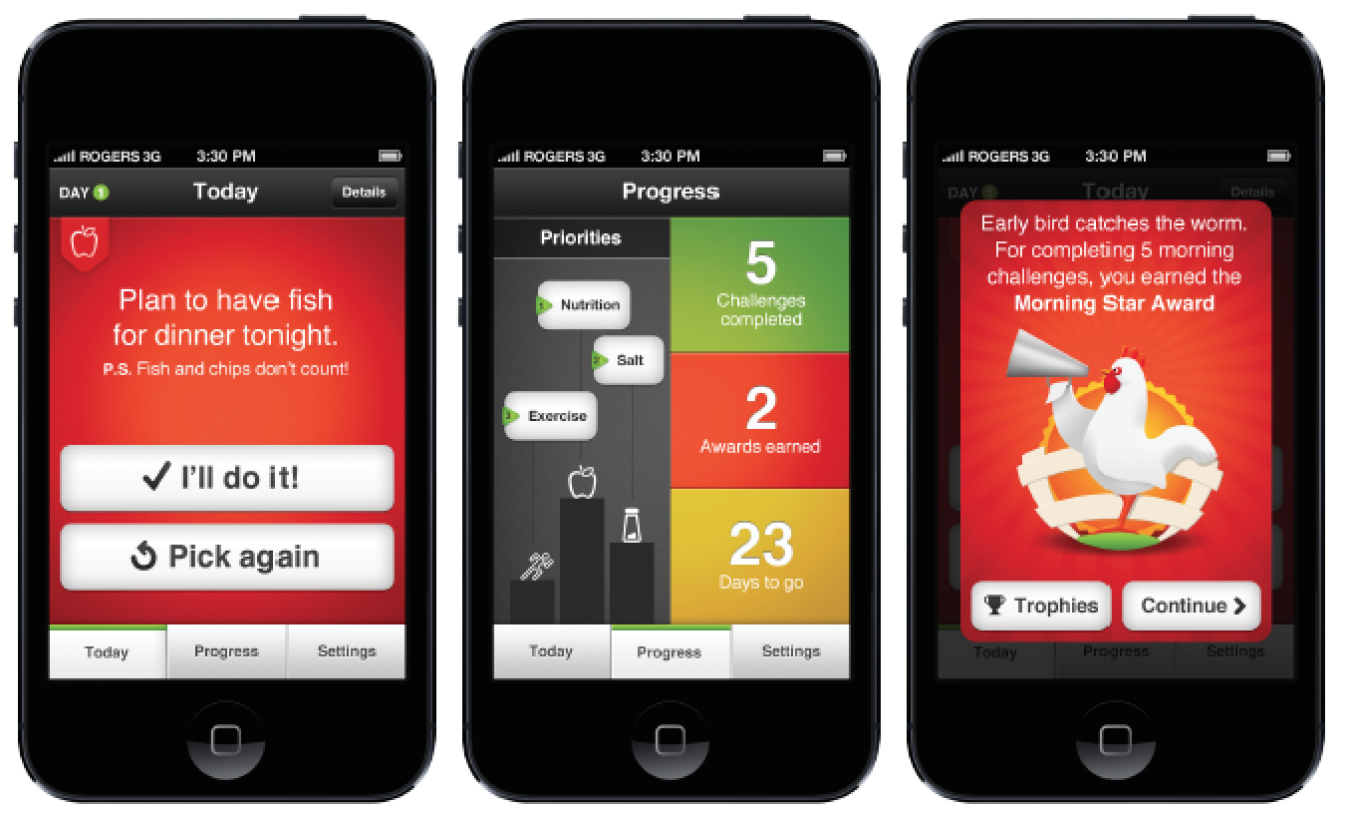
Components of the <30 Day mobile app for dynamic risk assessment for heart and disease and stroke. Left: users can either commit to the challenge or choose another one. Middle: users can review their risk factors and progress. Right: playful badges highlight various accomplishments throughout the <30 Day challenge.

On completing the risk assessment (see Figure 1 and Multimedia Appendix 1), users were presented with a list of their modifiable risk factors, which they could prioritize (first, second, and third) according to what they wanted to work on over the course of 30 days. The mobile app suggested simple daily activities based on identified risk factors, provided resources and encouragement, and tailored content to individuals’ risk profiles. When users were presented with a challenge, they had the option to *skip* the challenge and browse for more options or to *accept* the challenge. If they accepted the challenge, on the following day they would be asked whether they had completed the challenge, at which point they could proceed to select their next challenge.

To promote the completion of at least one heart health challenge per day, the app included an achievements system in the form of badges and a progress review module that was presented every 7 days. The Heart and Stroke Foundation also launched an incentive promotion for 1 month, where users were rewarded with AIR MILES points (AIR MILES Reward Program, LoyaltyOne, Co, Toronto, ON, Canada) for downloading the app. Previous studies have indicated that positive feedback in the form of a rewards system can motivate users to participate in self-management behaviors and improve health outcomes [13-15].

### Data Collection

The primary data source for this evaluation was anonymized usage data collected over 5 consecutive months. The data set consisted of responses to the health risk assessment and subsequent app usage, based on a unique installation of the app. Given that the risk assessment required demographic and anthropometric data, it was possible to evaluate app usage based on demographic and at-risk subgroups. Feedback from users was received through both the iTunes App Store and the technical support emails. Consent was acquired from all users through the mobile license agreement presented in the app on first use. The user group for this evaluation included all those who downloaded and used the app within the study time limits.

### Data Analysis

We created output files for data processing using Structured Query Language queries to the <30 Days app database. LabVIEW (National Instruments) was used to further process the data into a format acceptable by SPSS (IBM Corporation). We then ported the resulting data file into SPSS for statistical analysis.

Descriptive statistics helped identify usage patterns and the effectiveness of key features of the app. Independent *t* tests and chi-square statistics determined differences between groups, such as male and female groups, and incentives and nonincentives groups. We conducted between-subjects analysis of variance to analyze differences between age groups and engagements levels. A Pearson correlation analysis was conducted to assess the relationship between factors identified in the risk assessment and engagement levels. A multiple regression analysis on the number of challenges completed by the users was conducted to better understand the effect of the numerous independent variables on the uptake of the app and engagement levels.

Lastly, a thematic analysis was conducted on the comments received from users, where comments were compiled, organized, and assessed for recurrent themes. We derived major themes pertaining to the usability and utility of the app as critical feedback for improving the future versions of the app.

## Results

**Figure 2:**
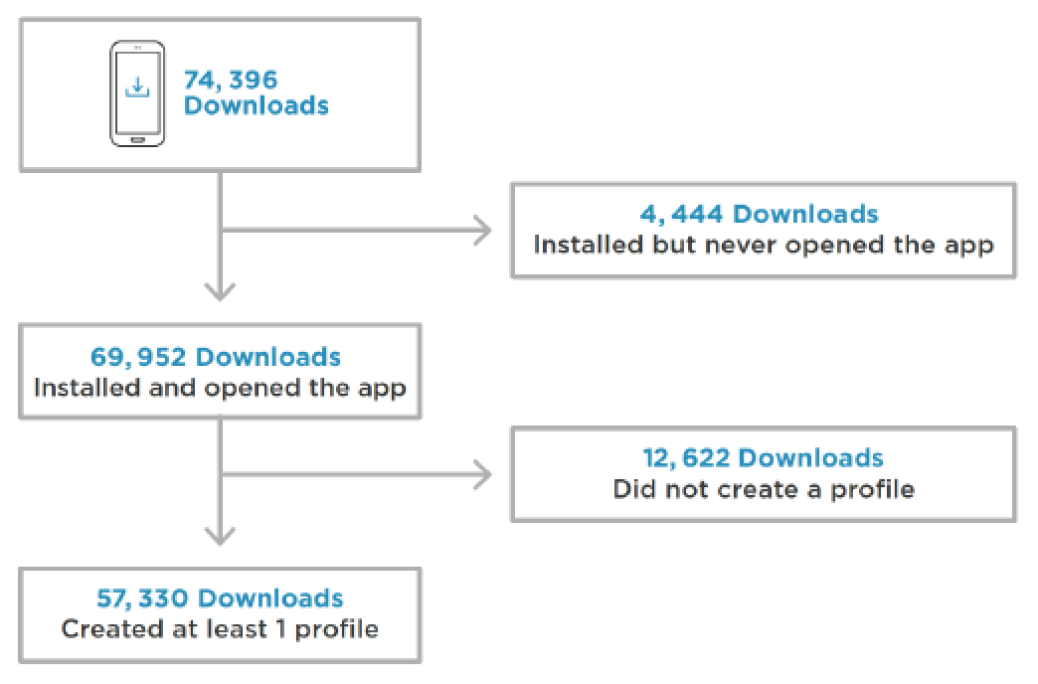
Number of users who downloaded and launched the <30 Days app and completed the risk assessment.

Of the 74,396 users who downloaded the app during the study period, 4,444 users installed the app but never launched it. Of the 69,952 users who downloaded and launched the app, 23,727 (33.92%) users registered during the AIR MILES promotion period, with 3,957 (5.66%) users who opened the app but did not complete the risk assessment. Altogether, 12,622 users never created a profile and consequently never completed the risk assessment. As Figure 2 shows, the final data set used for this analysis consisted of the data obtained from 57,330 users.

The AIR MILES incentive attracted 41.36% (6,184/14,950) of the total male users and 32.06% (13,586/42,380) of the total female users who downloaded the app and created a profile (n=57,330, χ^2^_1_= 423.7, *P*<.001, ϕ_Cramer_ = .086). The incentive engaged primarily users who were 31–70 years of years of age (χ^2^_8_ = 586.1, *P*<.001, ϕ_Cramer_ = .101). On average, users in the incentives group completed slightly more challenges during the first 30 days of the intervention (mean 7.9, SD 0.13) than those in the nonincentives group (mean 6.1, SD 0.06, *t*_28870_ = –12.293, *P*<.001, *d*=0.12, 95% CI –2.02 to –1.47). Additional data showed that during the promotion period, the overall number of downloads per day increased from 326 to 1186.

### Demographic Characteristics

As Table 1 shows, most users were female (42,380/57,330, 73.92%), between the ages of 21 and 30 years (19,200/57,330, 33.49%), and white (39,700/57,330, 69.25%). The uptake from the younger demographic was significantly higher than that from the older demographic, with only 8.75% (5018/57,330) of users aged ≥51 years downloading the app compared with 59.38% (34,042/57,330) of users aged 21 to 30 years. The average body mass index (BMI) was 28.0 (SD 5.2) for male and 26.9 (SD 6.5) for female users, classifying the majority of users as overweight (BMI 25–30 kg/m^2^).

**Table 1:** Demographics of users who completed the <30 Days health risk assessment app (n=57,330).

Characteristic		n (%)
**Sex**		
	Male	14,950 (26.08)
	Female	42,380 (73.92)
**Age group (years)**		
	≤20	14,842 (25.89)
	21–30	19,200 (33.49)
	31–40	11,464 (20.00)
	41–50	6782 (11.83)
	51–60	3777 (6.59)
	61–70	1125 (1.96)
	71–80	116 (0.2)
	81–90	13 (0)
	≥91+	11 (0)
**Ethnicity/race**		
	White	39,700 (69.25)
	Latin American	2374 (4.14)
	Chinese	2091 (3.65)
	South Asian	2057 (3.59)
	African heritage	1928 (3.36)
	Other	9180 (16.01)

### Modifiable Risk Factors

The most prevalent risk factor was poor nutrition (49,711/57,330, 86.71%), followed by lack of exercise (29,776/57,330, 51.94%), stress (28,592/57,330, 49.87%), excessive salt intake (17,333/57,330, 30.23%), tobacco use (8499/57,330, 14.82%), and excessive alcohol use (5950/57,330, 10.38%). On average, women had slightly more risk factors (mean 2.5, SD 0.01) compared with men (mean 2.3, SD 0.01; *t*_26,446_= –12.648, *d*=0.11, *P*<.001, 95% CI –0.16 to –0.12). Analysis of variance showed a relatively small but statistically significant difference in risk factors between age groups (*F*_8,57_ 321 = 142.55, *P*<.001, η^2^ = 0.02). More precisely, users in the 21–30 (mean 2.6, SD 0.01) and 31–40 (mean 2.5, SD 0.01) year age groups had a higher mean number of risk factors than those in the 61–70 (mean 1.8, SD 0.3) and 71–80 (mean 1.4, SD 0.9) year age groups.

### Health Conditions and Nutritional Habits

The risk assessment asked users to identify health conditions they might have had or health conditions of which they had a family history. Overall, 44.50% (25,511/57,330) of users reported having at least one of the listed conditions (depression, diabetes, heart disease, stroke, high blood pressure, high cholesterol, renal disease, and sleep apnea), and 31.12% (17,839/57,330) of users reported having depression or anxiety, as Table 2 shows. A large proportion of users (39,793/57,330, 69.41%) reported having a family history of diabetes or high blood sugar, heart disease, high blood pressure, high cholesterol, or stroke.

In terms of nutritional habits, the following percentage of users reported eating these foods 3 or more times a week: 45.34% (25,996/57,330) ate high-fat foods, 30.56% (17,520/57,330) ate fast food, 24.24% (13,898/57,330) ate foods rich in omega-3 polyunsaturated fatty acids, 42.37% (24,263/57,330) ate 5 or more servings of fruits and vegetables per day, and 17.95% (10,291/57,330) ate none of these.

### Engagement Levels

We selected a subsample of all users (52,431/74,396) who downloaded and opened the app, created a profile, and had at least 30 days to use the app for this part of the evaluation to ensure that all users had an opportunity to complete the challenges. As Table 3 shows, 84.94% (44,537/52,431) of users had very low and low levels of engagement, and 10.03% (5259/52,431) of users had high levels of engagement. The categorization and range of engagement levels were defined a priori by the Heart and Stroke Foundation, where a categorization of very low describes those who completed a profile but did not complete any challenges, and low, moderate, and high describe those who completed 1–14, 15–21, and ≥22 challenges, respectively.

**Table 2:** Health conditions identified by users who completed the <30 Days health risk assessment app (n=57,330).

Questions posed to users		Positive response, n (%)
**Do you have any of the following conditions?**		
	Depression or anxiety	17,839 (31.12)
	Diabetes or high blood sugar	1978 (3.45)
	History of heart disease	1736 (3.03)
	History of stroke	746 (1.3)
	High blood pressure	5564 (9.71)
	High cholesterol or triglycerides	4847 (8.45)
	Renal disease	209 (0.4)
	Sleep apnea	3611 (6.30)
	None of the above	31,819 (55.50)
**Do you have a family history of any of the following?**		
	Diabetes or high blood sugar	24,309 (42.40)
	Heart disease	15,916 (27.76)
	High blood pressure	25,204 (43.96)
	High cholesterol or triglycerides	16,678 (29.09)
	Stroke	10,806 (18.85)
	None of the above	17,537 (30.59)

**Table 3:** User engagement levels measured by the number of completed challenges in the first 30 days of using the <30 Days health risk assessment app (n=52,431).

Engagement level (challenges completed)	n (%)
Very low (0)	14,546 (27.74)
Low (1–14)	29,991 (57.20)
Moderate (15–21)	2635 (5.03)
High (≥22)	5259 (10.03)

**Figure 3:**
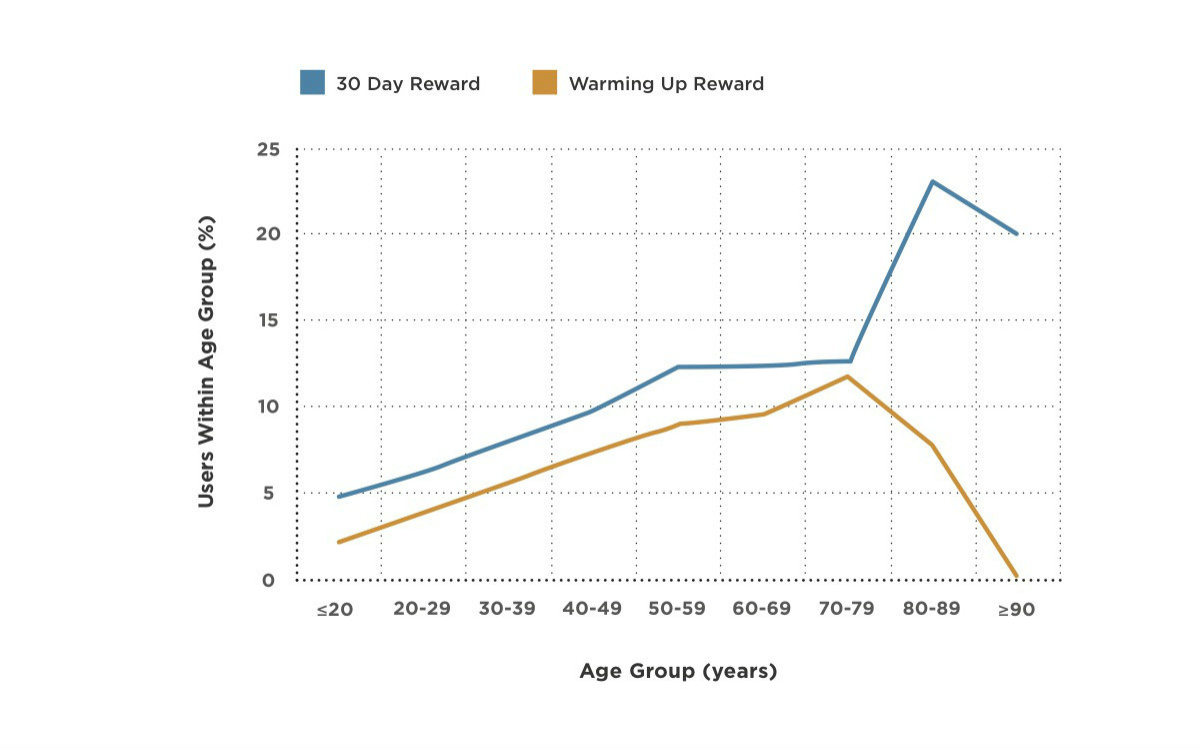
Rewards achieved in the <30 Days health risk assessment app by users per age group. This graph shows the distribution of users (n=52,431) who completed the health risk assessment and were part of the engagement subset.

We measured engagement by looking at the number of challenges each user completed in the first 30 days, according to the distribution presented on Table 3. One-sample *t* test indicated that engagement levels among female users (mean 7.10, SD 0.07, n=38,494) was slightly higher than those among male users (mean 6.73, SD 0.15, n=13,937; *t*_52,429_ = –2.509, *P*=.01, *d*=0.02, 95% CI –0.648 to –0.08). Analysis of variance showed a small, statistically significant effect of age on engagement levels (*F*_8, 52,422_ = 55.10, *P*<.001, η^2^ = 0.008), with older participants (>51 years of age) completing more challenges than younger participants (Table 4). The frequency of the virtual rewards (badges) offered to users through the app was also captured. Figure 3 illustrates that a higher percentage of individuals aged 50–70 years achieved rewards for consecutive use of the app over 7 days (Warming Up badge) and completing the 30- day (30 Day badge challenge).

As Table 5 shows, those who reported having health conditions, such as diabetes, heart disease, stroke, high blood pressure, and high cholesterol, completed more challenges. A correlation analysis revealed a positive but weak correlation between the number of challenges completed and the number of personal conditions (*r*=.025, *P*<.001) and the number family history conditions (*r*=.041, *P*<.001) reported.

We evaluated the effects of several potential predictors on the number of challenges through multiple regression analysis. Due to the large dataset, we tested the model against 14 predictors: sex, age group, ethnicity, height, weight, all 6 risk factors, incentive and nonincentive information, number of conditions, and number of family histories. The assumptions of independence of errors, linearity, unusual points and normality of residuals, and homoscedasticity were met. Of the predictors used in the regression, only sex, age group, ethnicity, 5 of the risk factors (all but alcohol), the presence of incentives, and the number of family histories predicted the number of challenges that the user completed (*F*_14, 56,538_ = 86.644, *P*<.001, adjusted *R* ^2^ = .021). Table 6 shows the regression coefficients and standard errors for each of the predictors.

**Table 4:** Number of challenges in the <30 Days health risk assessment app completed by age group (n=52,431).

Age group (years)	Number of challenges completed in first 30 days		n (%)
	Mean	SD	
≤20	5.44	11.08	13,291 (25.35)
21–30	6.47	12.23	17,571 (33.51)
31–40	7.67	18.11	10,604 (20.22)
41–50	8.59	16.71	6297 (12.01)
51–60	9.78	19.96	3485 (6.65)
61–70	9.74	16.10	1050 (2.00)
71–80	9.89	15.69	110 (0.2)
81–90	10.08	16.09	13 (0)
≥91	19.80	36.59	10 (0)

**Table 5:** Number of challenges that users completed based on whether they identified as having a personal or family history of various health conditions in the <30 Days health risk assessment app.

Health condition		Had condition			Did not have condition			*t*_52,429_ value	Cohen *d*	*P* value
		No.	Mean	SD	No.	Mean	SD			
**Personal**										
	Depression	16,112	6.97	15.59	36,319	7.01	14.25	0.25	–0.003	.80
	Diabetes	1795	7.87	14.32	50,636	6.97	14.69	–2.56	0.60	.05
	History of heart disease	1581	8.60	19.47	50,850	6.95	14.50	–4.42	0.11	<.001
	History of stroke	688	9.27	21.33	51,743	6.97	14. 56	–4.10	0.15	<.001
	High blood pressure	5079	7.93	14.24	47,362	6.90	14.72	–4.76	0.70	<.001
	High cholesterol	4429	8.24	22.19	48,002	6.88	13.77	–5.87	0.09	<.001
	Renal disease	191	7.95	14.63	52,240	7.00	14.68	–0.90	0.06	.37
	Sleep apnea	3254	7.40	14.78	49,177	6.97	14.67	–1.61	0.03	.11
**Family history**										
	Diabetes	22,177	7.25	16.05	30,254	6.81	13.58	–3.69	0.03	.01
	Heart disease	14,533	7.83	17.57	37,898	6.68	13.39	–6.24	0.11	<.001
	Stroke	9874	7.83	16.19	42,557	6.81	14.293	–4.096	0.15	<.001
	High blood pressure	23,063	7.43	15.52	29,368	6.66	13.97	–5.94	0.05	<.001
	High cholesterol	15,233	7.69	17.69	37,198	6.71	13.24	–6.939	0.07	<.001

**Table 6:** Summary of the multiple regression analysis of predictors of how many challenges would be completed by users of the <30 Days health risk assessment app.

Variable		B^a^	SE_B_^b^	Beta	*P* value
Intercept		2.741	0.855		<.001
Sex		1.064	0.154	0.033	<.001
Age group		0.911	0.051	0.083	<.001
Ethnicity		–0.074	0.021	–0.015	<.001
Height		0.006	0.004	0.007	.16
Weight		0.002	0.002	0.005	.37
**Risk factor**					
	Alcohol	0.233	0.199	0.005	.24
	Smoking	–0.761	0.171	–0.019	<.001
	Stress	0.641	0.126	0.022	<.001
	Exercise	–2.015	0.123	–0.071	<.001
	Salt intake	–1.174	0.132	–0.038	<.001
	Nutrition	–1.071	0.189	–0.025	<.001
Incentive (AIR MILES)		1.573	0.126	0.052	<.001
Number of conditions		–0.007	0.075	0	.93
Number of family histories		0.315	0.043	0.033	<.001

^a^B: unstandardized regression coefficient.

^b^SE_B_: standard error of the coefficient.

^c^Beta: standardized coefficient.

### Analysis of Challenges

**Figure 4:**
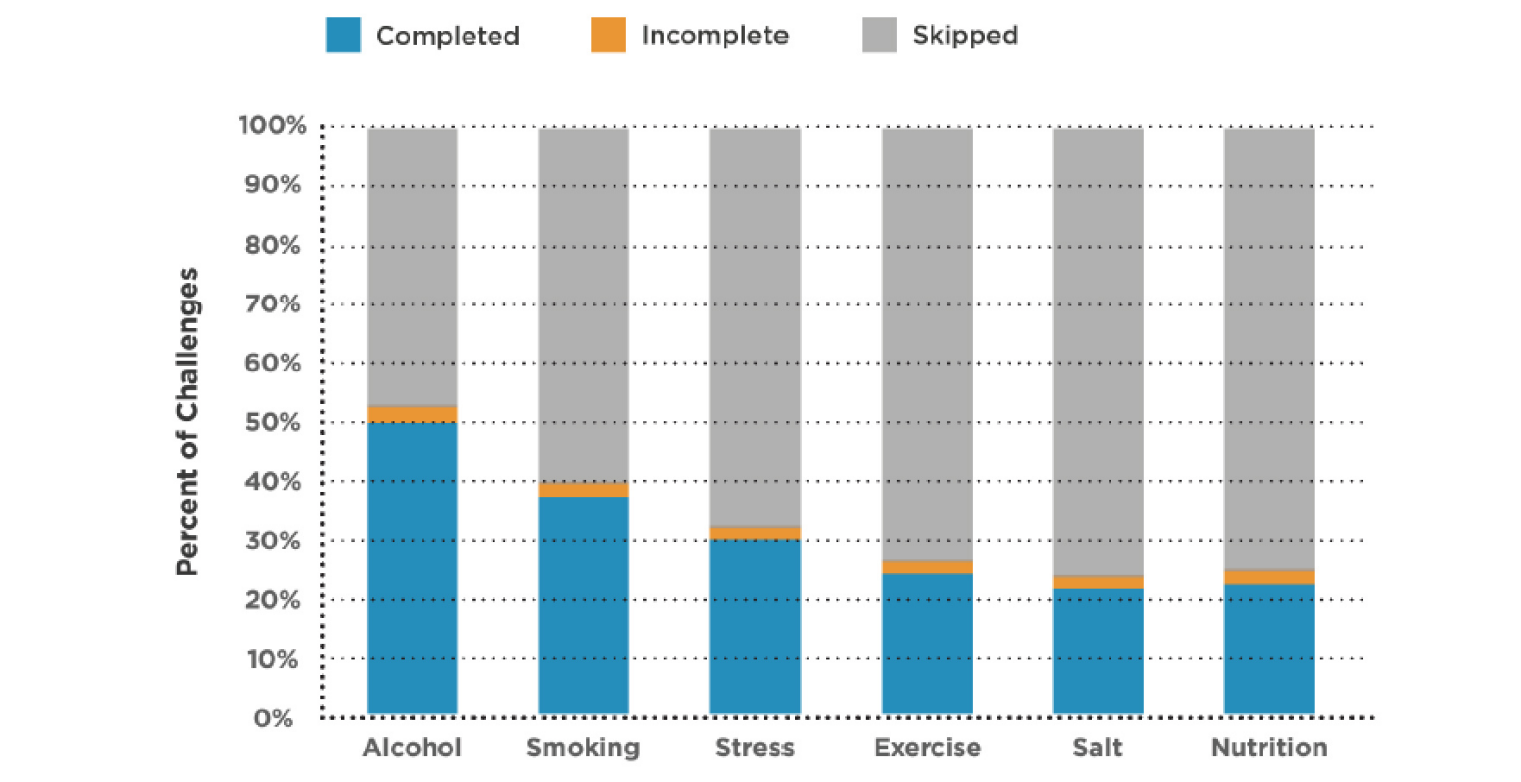
Challenge completion rates by risk factor by users of the <30 Days health risk assessment app.

When presented with a daily challenge, users have the option either to *skip* or to *accept* the challenge, and then later mark it as *complete* or *incomplete* the following day. Though 92.99% (464,097/499,078) of the *accepted* challenges were completed, a large majority (1,183,013/1,682,091, 70.33%) of the challenges presented to the users for selection were skipped*.* Figure 4 provides a breakdown of the completion rates per risk factor. Although nutrition was the most prevalent risk factor, the challenges from this category had the highest skip rate (546,850/731,010, 74.81%), with only 8.82% (64,475/731,010) of challenges completed. In contrast, 50.39% (59,460/118,006) of the alcohol challenges were completed, with 46.95% (55,400/118,006) of them skipped.

### User Feedback

Over the study duration, we received 37 user comments, with 24 comments submitted through the iTunes store and 13 comments through the technical support email. Many users (n=17) stated that the daily tips were a good way of incorporating attainable healthy changes into their daily routines and facilitating heart disease prevention. Another subset (n=5) mentioned that the challenges were not applicable to them because the challenges were more focused on urban lifestyles than on the rural setting.

## Discussion

Our analysis of the <30 Days usage data provided several insights into the uptake and effectiveness of a consumer-friendly mobile app for the engagement of populations in the management of cardiovascular risk. The majority of users were female and from the younger demographic (21-30 years old) group. However, the older user groups demonstrated higher levels of engagement and completed more challenges. We anticipated that the demographic would skew toward younger adults, who are characteristic of the population who own mobile phones and download mobile apps [16,17].

However, the finding that the level of engagement increased with age was unique. The Pew Research Centre’s November 2011 survey showed that younger users not only downloaded more apps but also tended to be 50% more likely than users aged ≥50 years to use the apps [16]. In this case, we can infer that the older user groups were more committed to their health goals and were potentially more willing to participate in daily self-management activities.

The multiple regression analysis suggested that sex, age group, ethnicity, having 5 of the risk factors (all but alcohol), the presence of incentives, and the number of family histories are predictors of the number of challenges completed by a user (*F*_14, 56,538_= 86.644, *P*<.001, adjusted *R* ^2^ = .021). The analysis also demonstrated that these variables explain only 2% of the variation in the number of challenges completed. The social phenomena around the use of apps for lifestyle modification are complex and multidimensional, consequently making it difficult to explain the large amount of variation. Other factors, such as the influence of the user’s environment, familial structure, socioeconomic status, and familiarity with technology, could have affected the number of challenges completed and the resulting low *R* ^2^ value [18]. Furthermore, those who identified having a diagnosis or family history of health conditions had higher levels of participation. This implies that individuals who are aware of their familial risk or who have a health condition may have a greater perceived susceptibility or risk and may be more inclined to use a risk management app than would those who have not yet been exposed to health-related problems [19]. A review by Imes et al [20] found that both the awareness of family history and perceived personal risk are necessary predictors of change, and when coupled with interventions that, first, are founded in theoretical frameworks and, second, provide lifestyle modification options, can guide individuals at risk toward improved health behavior change. Moreover, certain risk factors influenced the number of challenges that an individual completed. Physical inactivity, nutrition, and salt and tobacco intake had a negative association, demonstrating that users who had these risk factors completed fewer challenges than did users without these risk factors. Stress, on the other hand, had a positive association, and consequently users with this risk factor completed more challenges than did users without the stress risk factor. The negative association is counterintuitive, as one would expect that the presence of a risk factor would result in increased engagement with one’s health.

Engagement levels were comparable in the incentives group and the nonincentives group, which implies that a one-time monetary incentive could potentially trigger sustained motivation and engage individuals in a short-term preventive intervention. The AIR MILES incentives increased uptake among the male and the older demographics, suggesting that the reward form (cash, points, gift card, etc) and vendors could have an impact on attracting specific user demographics [21]. For example, are the majority of AIR MILES consumers older than the iTunes App Store user base, who may be younger? While evidence that financial incentives are more effective at changing behavior than usual care is increasing, the effect of alternative incentive types on uptake from target populations with varying sociodemographic settings remains unexplored [14,15].

Poor nutrition, physical inactivity, and stress were the most prevalent risk factors identified, coupled with the majority of users having a BMI between 25 and 30 kg/m^2^, suggesting that being overweight was a predominant risk factor among the users.

Furthermore, the feedback received in the form of comments from the users expressed the need for challenges that were relevant to their situation and needs. For individuals who work in the service industry, for example, getting up from their desk and going for a walk may not be relevant. Also, the high frequency of challenges skipped suggests that perhaps the challenges were not applicable to the user, forcing them to search for options. The individualization of content and selection of strategies based on personal preference can optimize engagement and have a significant impact on the effectiveness of the intervention [22,23]. Offering customization by presenting users with challenges tailored to their profile (age group, location, ethnicity, etc) could increase the number of appropriate challenges shown to the users, resulting in higher engagement and motivation levels.

### Limitations

The data obtained from both the risk assessment and the challenge completion rates were dependent on self-reporting, which could have influenced the overall accuracy of the data. Given that the app was made available to for iOS (eg, iPhone, iPad, iPod Touch; Apple, Inc) users, the usage data do not fully represent all consumers, specifically Android users, who now account for the majority of the mobile phone market [17]. Mobile phone ownership varies by socioeconomic background, and people with higher levels of income and education have been shown to be more likely to own iPhones [17].

### Conclusions

While the younger population downloaded the app the most, the older population demonstrated greater and sustained engagement levels. Behavior change apps have the potential to reach a targeted population previously thought to be uninterested in or unable to use mobile apps. However, lifestyle modification and risk reduction tools must be useful, relevant, and integrated into daily routines, where the opportunity to change behavior significantly is the greatest. Mobile devices may be an effective channel for delivering such interventions to populations in various settings. The development of behavior change mobile apps should assume that older adults will engage if the behavior change elements are suitably designed, integrated into daily routines, and tailored to their needs.

While incentives may be the stepping-stone that is needed to guide the general population toward preventive tools and promote maintenance of positive behavior change, the incentive type and its influence on specific user groups needs to be further explored. It is clear, however, that capturing population-level usage information offers tremendous insight into user behaviors and interactions. The possibility of expanding the data collection beyond a single intervention, and capturing data from peripheral devices and the environment, or even linking such findings with long-term outcomes, could better inform the development, delivery, and uptake of preventive tools.

## Appendix

Multimedia Appendix 1 The <30 Days health risk assessment questions.

## Abbreviations

BMI: body mass index
CVD: cardiovascular disease
