# Supplementary material for: Uptake of a Consumer-Focused mHealth Application for the Assessment and Prevention of Heart Disease: The <30 Days Study
Source: JMIR Mhealth Uhealth. 2016 Mar 24;4(1):e32. doi: 10.2196/mhealth.4730 (PMC4824871; doi:10.2196/mhealth.4730)
Supplement: Multimedia Appendix 1 [file mhealth_v4i1e32_app1.pdf]

Multimedia Appendix 1

Table A. <30 Days health risk assessment questions.

Question	Answers
**Unmodifiable Risk Factors**	
What is your age?	(User selects age)
What is your ethnicity?	African heritage /Arab /Caucasian /Chinese /Filipino/ South Asian (e.g. Indian, Pakistani)/ Southeast/Asian (e.g. Vietnamese)/ West Asian (e.g. Iranian)/ Other
Do you have a family history of:	Diabetes or high blood sugar /Heart disease/ High blood pressure/ High cholesterol or triglycerides/ Stroke/ None of the above
Do you have any of the following conditions?	Depression or anxiety/ Diabetes or high blood sugar/ History of heart disease/ History of stroke/ High blood pressure/High cholesterol or triglycerides/ Renal disease/ Sleep apnea/ None of the above
**Modifiable Risk Factors**	
What is your height?	(User selects height)*
What is your waist measurement?	(User selects height)*
Do you eat the following 3 or more times a week?	High fat foods (e.g. fatty meats, donuts)/Fast food (e.g. hamburger, French fries)/Foods rich in omega-3 (e.g. cold-water fish such as salmon)/5 or more servings of fruits and vegetables a day/None of the above
Are you moderately active for at least 3-60 minutes during 4 or more days of the week?	Yes/No
How often do you feel overwhelmed by stress?	Very often/Not too often
What's your salt intake like?	I love salt!/I limit my salt!
Do you smoke?	Yes/No
Do you drink more than 1-2 drinks containing alcohol a day, or more than 10 drinks a week?	Yes /No
